# Retrospect of the Autopsies Performed in the Vienna Lunatic Asylum of the Patients Who Died in 1853

**Published:** 1855-10-01

**Authors:** 

**Affiliations:** Second Physician to the Institution.


### RETROSPECT OF THE AUTOPSIES PERFORMED IN THE VIENNA LUNATIC ASYLUM OF THE PATIENTS WHO DIED IN 1853.

BY DR. GAUSTER,

*Second Physician to the Institution.*

THE physiology and pathology of the nervous system are the basis, the nucleus of psychiatry. A disease can only be caused by a diseased organ: a Power, the Immeasureable, the Imponderable, the Transcendental, cannot be sick! In the new Vienna Lunatic Asylum the end has been steadily kept in view of tracing the material appearances, without overlooking the rest.

Statistical tables play at the present moment no inconsiderable part: perhaps they are too much confided in. A statistical collection of our autopsies could not in prudence warrant any important results, because they have not yet been accompanied by microscopical investigation. But we may still hope for some negative replies, and even this is a gain. Nervous physiology and pathology have as yet but little of the positive. The *post-mortem* examinations of the year 1853 gave a negative answer to the question—Can a given form of psychical disease be traced back with scientific exactness, or with probability, to particular pathologies—anatomical changes of the organs concerned? But even here, in the gloom of the comfortless though some rays of light are revealed which promise that investigation will lead to definite results in the future, it is a source of satisfaction, at least, that what science has already formuled, will be found to be confirmed by our conclusions.

179 patients died in the year 1853 in the Vienna Imperial Lunatic Asylum. Of these 171 were submitted to inspection.

In order to determine the relations of the individual phenomena to each other, the principal phrenopathic conditions have been compared with the most remarkable appearances in the brain, as well as with those of the organs of circulation; and lastly, it has been sought how often they existed without alterations in the brain. Since it is known what great influence disturbances of the circulation of the blood exert over the causation, development, and persistence of phrenopathies, there must also be taken in careful consideration thickened and calcareous arteries at the basis of the brain, dilatations, and deposits of the aorta, especially of the arch and ascending portion; and lastly, diseases of the heart, as hypertrophy and insufficiency of the valves.

The organs of digestion have been passed over, because their diseases, in a phrenopathic point of view, are sometimes quite absent, and at others unimportant. So it is with the liver, which, although not seldom having some influence upon mental alienation, is yet too often judged and condemned unjustly.

The organs of respiration also have not been much considered. These comparisons of the most important particulars have not yet given any remarkable results; but it cannot be doubted that the origin of individual forms of alienation, contemporaneously with different diseases, and not unfrequently with several together, is possible, just as we find to be the case with functional disturbances of other kinds.

It cannot be denied that the result of the comparison of the appearances in the dead body with the observation of the living man will appear of greater value, if we compare not only the forms of the disease, but also the symptoms of the diseases. But this is a work much more laborious, and one demanding the closest application and continuous observation.

The per-centual proportions of the more important results of the *post-mortem* inspections have been compared with the gross number of the inspections, and with the individual forms of mental diseases.

The fundamental forms of alienation in vogue have been used: the forms of exaltation, mania, delirium tremens; of depression, melancholy in its various aspects, and those of debility; secondary dementia, dementia with progressive paralysis, congenital dementia.

The most important deductions from the whole comparisons are the following:—

1. The highest per-centage upon all the *post-mortem* appearances is exhibited by œdema of the membranes, 52·046 per %; chronic hydrocephalus, 50·292 per %; œdema of the substance of the brain, 23·946 per %; sclerosis of the brain, 21·637 per %; diseases of the aorta, 12·869 per %; thickening of the inner membranes of the brain, 11·111 per %; diseases of the heart, 9·941 per %. No abnormal appearance in the brain was found in nineteen cases, or 11·111 per %. Here it is to be observed that every condition of greater hardness of the brain-mass was estimated as sclerosis. Very strongly-marked sclerosis was present in two cases only.

2. There was no one of the more frequently found brain-appearances that was not observed in connexion with all the fundamental forms of alienation, excepting the adhesion of the meninges with the periphery of the brain, which was only found in the exaltation-forms, in conditions of debility, and of epilepsy with mental disturbance, preponderating, however, in paralytic dementia; then the thickened and calcareous arteries of the basis of the brain which was wanting in the forms marked by depression; the discoloration of the grey substance, heart-diseases and the aortal diseases, all of which were found wanting in epilepsy with mental disturbance.

3. Instances occurred in all the chief forms of alienation, in which no disease was found in the brain, but most frequently was this the case in the forms of depression, 21·739 per %; least frequently in the exaltation-forms, 4·878 per %.

4. Most commonly serous effusions were found, especially on the surface of the brain, then in the cavities; serous effusions in the meninges were most frequent in the exaltation-forms, 68·293 per %; thence falling in the depression-forms to 60·869 per %; in conditions of debility to 53·763 per %; in epilepsy with mental disturbance to 50 per %. Serous effusions into the cerebral cavities were also found most frequently in the exaltation-forms, 60·975 per %; then in conditions of debility 52·688 per %; least often in the depression-forms, 26·087 per %. Œdema of the brain-mass was also most frequent in the exaltation-forms, 26·831 per %; and least frequently in epilepsy with mental disturbance, 21·429 per %.

5. The greater hardness of the brain, estimated here under the designation sclerosis, was most frequently found in the depression-forms, 30·435 per %; least so in the exaltation-forms, 14·634 per %.

6. Thickening of the arachnoid was most frequently found in the exaltation-forms, 14·634 per %; then in epilepsy with mental disturbance, 14·286 per %; least so in the depression-forms, 4·348 per %.

7. Bony formations on the skull and in the brain were not found in the depression-forms. Those on the skull were most frequently seen in the exaltation-forms, 7·317 per %; and in epilepsy with mental disturbance, 7·143 per %; those of the meninges in conditions of debility, 5·376 per %; chiefly in paralytic dementia; then these were found in the exaltation-forms, 4·878 per %.

8. The discoloration of the grey substance was most common in the exaltation-forms, 7·317 per %; then in the conditions of debility, 6·452 per %; least so in the depression-forms, 4·348 per %; and in epilepsy with mental disturbance, never.

9. Diseased basal arteries and aorta were most common in the conditions of debility; the first, 9·677 per %; the latter, 15·054 per %; heart-diseases in the depression-forms, 13·043 per %; in exaltation-forms, 12·219 per %.

10. Uterine polypi and fibroid tumours were present in the exaltation-forms.

11. Diseased conditions of exhaustion, of weakness, as general anæmia, marasmus, tabes, were found most frequently in the conditions of debility, then in melancholy.

12. Tuberculosis plays especially a not inconsiderable part; excepting in epilepsy with mental disturbance, it was most common in the exaltation-forms.

13. Diseases of the bronchial tubes, as catarrh, bronchitis, bronchiectasis, were most common in the exaltation-forms.

14. Liver and stomach diseases were proportionately more frequent in delirium tremens: on the other hand, in the depression-forms many diseases of the kidneys and spleen were present.

15. In respect of the determinate pressure of one brain-appearance with another brain-appearance, or with a morbid appearance of the other organs, this analysis gives nothing certain or positive.

16. Lastly, no definite and incontrovertible conclusion as to the psychical disturbance present during life is indicated by the forms of morbid appearances; neither by combinations of these.

We will relate briefly some of the more interesting appearances:

1. A case of congenital partial absence of the left hemisphere of the brain, in an idiotic girl of thirteen, who died with dropsy and paralysis.

2. A case of absence of the corpus callosum in a clergyman, aged twenty-five, who died of tabes; and who, from his twentieth year, was epileptic after a fright, and then gradually became demented.

3. A case of fibrous carcinoma in the septum and fornix, extending to the substantia perforata and optic nerves, then losing itself in the cerebral substance above, and below, and behind, extending to the hippocampus major. The hinder part of the left lobe of the brain was yellow and softened; on the inner wall of the left posterior horn, on the border of the before-mentioned softened spot, the carcinoma was as large as an egg, and was adherent to the thalamus opticus of this side, and as well as the superficial laminæ of this last, was infiltrated with hæmorrhagic spots. The corpora striata and thalami of both sides were dragged out lengthwise. The patient had suffered from dementia following upon headaches and excitations.

4. A remarkable case of a foreign body in the brain-substance in a person thirty-four years old, who died in an epileptic fit. In the hairy scalp, three inches above the left ear, was a white bald spot; under the occipito-frontalis muscle there was found in the skull a cavity surrounded exteriorly by an edge somewhat rounded, and interiorly sharp, with a soft brownish callus adhering to the margin of the opening. After removal of the skull-cap there projected over the first opening, which was exactly over the interior branch of the middle meningeal arterial, into the dura mater, a black-brown body adhering to the fore-part of the callus, and penetrating into the brain, and just over the middle of the Sylvian fissure covered with a yellowish delicate callosity. Besides this there was present hyperæmia of the brain and meninges. The patient from his youth suffered from epilepsy; he had been struck three or four years back in a pot-house, nothing more being learned of him; he exhibited in the asylum dementia, with occasional excitations and epilepsy, but no symptom that pointed with clearness to their appearance was discovered.

5. A case of tubercle in the right hemisphere, in a patient who had suffered from dementia with hardened brain-mass, œdema of the brain and meninges, depositions in the aorta and pleuritic exudations. The tubercle was of the size of a walnut enclosed in the outer part of the right corpus striatum, by a reddish vascular web; in its outer layer it consisted of a yellow very thick mass, whilst the central part consisted of an œdematous cellular tissue. The surrounding structure was in a state of white softening, the rest very hard.

6. A case of hypertrophy of the thyroid gland, with œdema and thickening of the inner membranes of the brain, adhesion of these to the peripheral substance of the brain, and chronic hydrocephalus, then pulmonary tuberculosis. The thyroid gland was enlarged sixfold, its right lobe was so lengthened downwards that its rounded lower edge reached into the cavity of the chest down to the basis of the pericardium. The left vena anonyma was compressed by it, and quite closed; above the compressed spot, it and its branches were filled with coagula. The patient suffered from dementia, with strong hallucinations, excitation, and progressive general paralysis.

In conclusion, we append some remarks upon the results of this analysis, and especially in comparison with those found elsewhere.

When Guislain, in his latest work, says: "In spite of my many and painstaking investigations on the bodies of insane after the elucidation of the nature and seat of mental diseases, I must confess that I have not arrived at the hoped-for results," this admission of so great an authority will excuse the slender results we now publish. But since no doubt at present exists that phrenopathies consist in a disease of the nervous centres, this observation will only stimulate us to continued and closer toil, and to employ every possible aid. Moreover, the continued investigation has ever shown fewer cases of insanity in which no abnormal brain-appearance was found.

For example, Pinel found, out of 261 autopsies, 68; Esquirol, out of 277, only 77 cases in which diseases of the brain were recognised in the body. On the other hand, Parchappe found 152 such cases out of 160 autopsies; and Webster, out of 72 autopsies, alterations in the brain in every case. In our report, there appear out of 171 autopsies, 19 cases of absence of abnormal brain-appearances; and it is to be remarked that the microscope was not employed.*

The most pre-eminent part in the preceding analysis is played by serous effusions into the brain and its meninges. It is often difficult, indeed, to determine whether these be primary or secondary, or what is their importance in relation to the form of disease. There is an old controversy, whether serous effusions primarily act upon the psychical functional phenomena through pressure, or secondarily proceeding from phrenitis, or habitual congestions, only keep up the scene. Later times established both views. This statement shows that they are found in all the forms in all congestions and inflammations. The hardness of the brain, as here understood, showed itself relatively highest in the depression-forms. Induration of the brain, according to Guislain, was found 25 times in 100. This analysis shows, out of 171 autopsies, 37 cases, or 21·637 per $\frac{0}{0}$, although the extent varied much. Griesinger thinks induration belongs especially to dementia.

Morgagni found, in 13 autopsies, 11 times sclerosis, especially of the medullary substance. Parchappe has, in 313 insane persons, found 81 times the entire brain-medulla sclerosed; Esquirol found, in 54 demented persons, 15 times increased hardness of the medullary substance of the brain.

The adhesions of the membranes to the periphery of the brain have given rise to many discussions. By some it has been held that congestions in the cortical substance or pia-mater; by some, that inflammations of those structures, were the cause; by some they are regarded as the result of organized exudations. It has been thought that the most superficial layers of the cortical substance were destroyed through them. Recent researches give to both the two first views great probability. Their presence in the conditions of debility is frequent, 11·828 per $\frac{0}{0}$: 11 times they were present in these forms of disease, and, moreover, 9 times in progressive paralysis. This agrees with the observations of Bayle and Parchappe.

* It is also to be remarked and to be regretted that the method of testing the absolute weight and the specific gravity of the brain, which has yielded such valuable results to Drs. Sankey, Bucknill, and others, was not employed. Let this be one of the "further possible aids" which the author promises to use in his future researches. The author, for example, does not appear to have recognised such a condition as atrophy of the brain—one of the most common and important of the conditions found in the brains of the insane.

Atrophy of the brain has been much discussed of late. Means of determining this condition with accuracy are wanting. Doubtful instances of atrophy were found four times.

Thickening of the inner membranes with opacity is a "condition which doubtless depends upon preceding congestion or inflammation," and perhaps upon other conditions yet unknown to us. We found these most frequently in the exaltation-forms, namely, out of forty-one cases, six times. In fourteen cases of epilepsy, twice.

Organic diseases of the heart, in connexion with alienation, have of late attracted much attention. Their relations have become of greater importance, inasmuch as heart-diseases have been disproved in remarkable frequency in living lunatics; and in these cases it ought not to be overlooked that greater excitation has attended unhealthy heart-sounds, which have been replaced by healthy sounds at periods of quietude. This has been observed in no small number of our patients. It cannot be denied that heart-diseases, through the "consequent irregular blood-circulation," may often occasion diseases of the brain and mental disturbance. The thickening and depositions of the basal arteries may often be the cause of brain-diseases, which induce disturbance or weakness of the intellectual force.

Tuberculosis has also given its contingent in this inquiry.

The author concludes by remarking that the post-mortem examinations were not conducted with all desirable minuteness, the arrangements of the dead-house of the hospital not permitting.

---

